# Genomics of stroke recovery and outcome

**DOI:** 10.1177/0271678X251332528

**Published:** 2025-04-11

**Authors:** Arne G Lindgren

**Affiliations:** Department of Neurology, Skåne University Hospital; Department of Clinical Sciences Lund, Neurology, 5193 Lund University, Lund, Sweden

**Keywords:** Genetics, genomics, stroke, recovery, outcome

## Abstract

The understanding of genomics has improved tremendously during the last decades. The concept of recovery and outcome after stroke has also progressed during this time. However, the connection between genomics and stroke recovery has only begun to emerge in a more structural and comprehensive way. Different types of outcomes and recovery occur after stroke. This depends on domain of neurological deficit, severity, resilience, receptivity for rehabilitation measures, and concomitant morbidity. Methods for assessing stroke patients’ prognosis depend on these factors. Different genetic approaches are possible and there is an increasing need for linking genetic findings to other omics as well as to clinically meaningful results. This review addresses recent advances and views in clinical genomics of stroke recovery and outcome in humans with focus on current and previous studies, concepts, and future perspectives.

## Why is genomics of stroke recovery and outcome important?

The possibilities for treating acute stroke have improved dramatically during the last decades. Recanalization therapy with thrombolysis and/or thrombectomy is now provided for many patients with acute ischemic stroke and the treatment of acute hemorrhagic stroke including blood pressure treatment and in some cases surgery has evolved considerably.

Despite this, only a minority of acute ischemic stroke patients receive recanalization treatment and individuals with ischemic or hemorrhagic stroke often have considerable deficits after the acute phase. Therefore, there is a great need for identifying new therapies for improved recovery after stroke.^1^

In clinical practice it is often difficult to determine the prognosis for the individual stroke patients. Even though on average, there is an improvement of function over time after the acute stroke onset, this average usually hides several individual courses that are not resembling the average recovery.^2,3^ Some patients continue to have severe deficits for long time periods whereas other improve substantially and rather unexpectedly. It is likely that genetic factors contribute to explaining some of this diversity in stroke recovery. Genetic variations may influence several biological mechanisms involved in stroke recovery including excitotoxicity, inflammation, apoptosis, plasticity, response to rehabilitation measures and pharmacological treatment, and resilience. For each of these mechanisms an understanding of genetic influence on other omics and metabolic pathways has a potential to result in development of improved therapies that are specific for each patient leading to individualized treatments regarding e.g. rehabilitation measures, and pharmacological treatments.

## Previous studies

Several reviews have addressed genomics and stroke recovery. In 2016, a review discussed the more general concepts of stroke recovery genetics.^4^ Since then, other reviews have focused on separate specific topics in stroke recovery. A recent review comprehensively listed candidate gene studies in ischemic stroke recovery.^5^ Another review listed genetic findings related to hemorrhagic stroke recovery.^6^ Different aspects on understanding how genetics can be used for understanding biological mechanisms in stroke outcome and recovery have been addressed.^7,8^ Reviews and studies have also focused on specific types of deficits, such as upper limb motor function,^9–11^ and cognitive function,^10,12^ emphasizing the importance of adequate and detailed phenotyping.^13^

However, the era of stroke recovery genetics is still in its infancy and more studies are needed. Signals may be small and from the individual patients’ perspective not fulfilling criteria as being clinically meaningful.^14^ But also such small signals may be of great importance as a proof-of-concept, indicating biological pathways that may be implemented in future therapies. Harmonization of phenotyping between cohorts is essential to obtain large enough samples to detect these small signals.^13^

Illustrations showing how different types of biological mechanisms depend on time after stroke onset have been published.^7,8,15^ and helped to improve models for understanding stroke recovery, from the very acute phase to the long-term chronic phase and provide support for defining new studies to obtain more answers on how recovery may occur.

## Stroke outcome and stroke recovery

**Figure 1.**
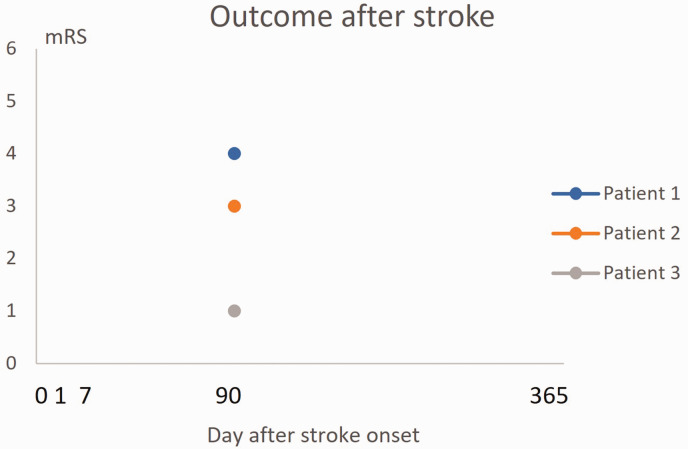
Outcome after stroke can be defined as the situation at a certain time point after stroke onset, not considering the severity of the acute phase deficits. Here is an example of mRS at 3 months for 3 imaginary patients. The figure provides no information about initial stroke severity. It is therefore unclear if any, and if so, how much, recovery has occurred after stroke onset. mRS indicates Modified Rankin scale.

In a strict sense, the term *stroke outcome* can be seen as describing the measurement of a condition at a certain time point after stroke without considering the status of the patient before this measurement (Figure 1). This makes it impossible to evaluate whether improvement or deteriorations has occurred.

**Figure 2.**
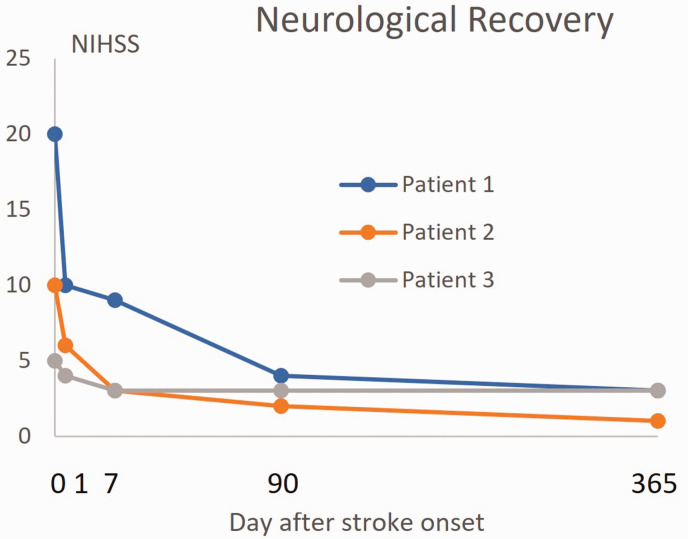
Recovery after stroke can be defined as changes between the acute phase of stroke onset and outcome at later time points. Example of 3 imaginary patients where NIHSS is evaluated at baseline, day 1, day 7, 3 months, and 1 year after stroke onset. Even though patient 1 had more severe neurological deficits at stroke onset, the recovery was much more pronounced than in patient 3 that proportionally recovered less regarding neurological function. NIHSS indicates NIH Stroke Scale.

The definition of *stroke recovery* on the contrary tries to incorporate the dynamic change over time after stroke. This needs consideration of the initial severity of stroke and even the pre-stroke condition for understanding if any, and if so, to what degree an improvement has appeared. One example is how the results from assessment according to the NIH Stroke Scale (NIHSS) may evolve over time (Figure 2). From a general perspective, the long-lasting recovery is the most important result because it indicates the new, more permanent situation for the patient after a stroke. Even so, immediate changes after stroke onset are of great importance because this period provides a time window for dramatic influences – positive^2,16,17^ or negative^16^ both with and without intervention^17^ – on the acute cerebral injury.

## Phenotyping - measuring stroke recovery – when and how

The preferred domains for evaluating stroke recovery are related to time after stroke onset because biological mechanisms and possibilities for assessment vary over time post-stroke. The Stroke Recovery and Rehabilitation Roundtable taskforce suggested dividing time periods after stroke into hyperacute (0–24 hours), acute (1–7 days), early subacute (7 days–3 months), late subacute (3 months-6 months), and chronic (beyond 6 months) phases (Figure 4).^15^ However, these time periods may vary depending on type of stroke (ischemic vs. hemorrhagic) and type of neurological deficits (e.g. motor, vs. language, vs. visual, vs. cognitive). Also, from the perspective of recanalization treatments in acute ischemic stroke, it may sometimes be useful to prolong the definition of hyperacute phase after stroke onset because a proportion of these patients may have delayed improvement occurring later than 24 hours,^18^ and it has been suggested that NIHSS at discharge (median 3 days, IQR 2–6 days)^19^ or at 7 days after stroke onset^20,21^ may be better tools for assessing subsequent stroke recovery between the acute and subacute phases.

Because genetic studies of stroke recovery are likely to detect relatively small signals, large datasets are required to obtain sufficient numbers of subjects. This is usually not possible within a single institution and therefore co-operation between collaborators in multi-center studies and consortia is needed. To facilitate data-handling and increase data quality, standardization and harmonization of phenotyping including outcome measures are crucial to enable meta-analyses and to increase the likelihood of detecting significant findings. The International Stroke Genomics Consortium (ISGC) and its branch, the ISGC Global Alliance Acute and Long-term Outcome studies in stroke (https://genestroke.wixsite.com/alliesinstroke) have published recommendations for phenotyping in stroke outcome and recovery studies and following these guidelines is recommended.^13^ These recommendations provide guidance regarding variables to focus on in acute studies and in long-term studies, as well as suggested minimal variable data sets for situations where research resources may be limited.^13^

### Outcome measures after the hyper-acute phase

The domains to measure recovery can be grouped into different categories with increasing complexity. These evaluation categories can be divided into blood or other tissue biomarkers, neuroimaging biomarkers, detailed (single domain) clinical evaluations, more global (often with sums of individual assessments) clinical evaluations, recurrent stroke, and survival (Table 1).

**Table 1.** Different measures can be used from more basic to more complex categories for evaluating genetic influence on stroke recovery. Several of these categories can be related to The International classification of Function, Disability, and Health.^22^

Category	Example	Comment	Example time period after index stroke
Biomarkers	Neuroimaging, e.g. Cerebral edema, lesion volume, fMRI, DTIBlood or other tissue samples for different omics		Neuroimaging edema: Hyper-acute, acuteSeveral neuroimaging methods also of interest for subacute and chronic time periodsBlood samples: all time periods
Body function and structures	NIHSS, MoCA	Subitems from e.g. NIHSS may be even more informative	NIHSS: all time periods; MoCA: Sub-acute, Chronic
Activities	Barthel index	Often global scales	Sub-acute, Chronic
Participation	mRS^a^	Often global scales	Sub-acute, Chronic
High level life categories/Health	EQ5D	More complex concepts, including PROMs	Sub-acute, Chronic
Recurrent stroke or new covert cerebrovascular incident	New clinical stroke being reported. Neuroimaging	Influenced by factors not directly involved in recovery after index stroke	All time periods
Survival	Information directly or from population registers	Influenced by factors not directly involved in recovery after index stroke	All time periods

fMRI indicates functional Magnetic Resonance Imaging; DTI: Diffusion Tensor Imaging; NIHSS: NIH Stroke Scale; MoCA: Montreal Cognitive Assessment; mRS: modified Rankin Scale; EQ5D: Euro-QoL-5D; PROMs: Patient Reported Outcome Measures.

a Especially mRS levels 1, 2, 3.^75^

The International Classification of Function, disability, and health (ICF)^22^ provides a concept where Body function, Activity, and Participation denote an increased complexity of the domains measured. *Body function* indicates the least complex physiological functions of individual body systems, e.g. simple motor, sensory, or visual functions. *Activity* denotes the execution of a task or action, where a combination of simple functions leads to purposeful actions e.g. lifting a cup, reading a text, or walking. *Participation* includes performing an activity as part of an involvement in a life situation - e.g. walking to a specific destination or using the reading of a text - to be part of an activity in interaction with other people.

Other important outcome measures, not included in the ICF concept, but with a possibly even higher level of complexity, include the psychological state, perceived quality of life, and satisfaction of the subject which may from the patients’ perspective be considered as the ultimate, most significant and meaningful domains. These usually require the patients’ intellectual and cognitive co-operation for assessment. This contrasts with biomarkers such as neuroimaging and blood sample analyses, discussed below, that usually do not need advanced co-operation from patients.

Many studies on stroke recovery have used evaluations similar to that in acute ischemic stroke treatment trials, i.e. more global measures where the modified Rankin Scale^23^ (mRS) at 3 months after stroke onset has been the golden standard. For clinical trials this has been regarded as a clinically meaningful^14^ measure and allowed for comparisons between trials. However, because the higher level domains such as the mRS often require a combination of lower level functions, it may be less likely that genetic variations influencing stroke recovery have a direct effect on these global domains and it has been questioned if it is optimal for stroke recovery genomic studies,^3^ to use global evaluation approaches resembling those in therapy trials.^24^ It may therefore be more rewarding to measure less complex body functions in genetic studies of stroke recovery to first demonstrate proof-of-concept for a biological mechanism before attempting to show genetic influence on higher more complex clinical levels.

**Figure 3.**
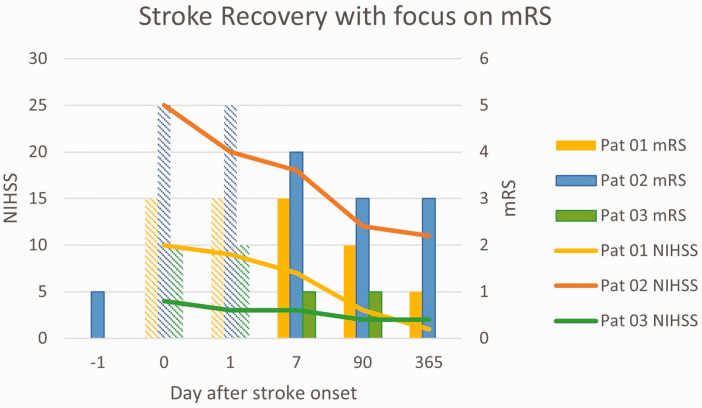
Relation between NIHSS and mRS in three imaginary stroke patients. NIHSS can be assessed at stroke onset and later. In contrast, mRS is difficult or impossible to assess during the initial hours after the acute onset and also during the initial days, and is therefore shown with less intense color in the figure. Pre-stroke mRS is of interest to understand the impact of the index stroke. Patient 01 and patient 03 had pre-stroke mRS = 0. mRS indicates modified Rankin Scale; NIHSS: NIH Stroke Scale.

The type of cerebral damage also has important implications for recovery and there are differences between ischemic and hemorrhagic injuries as well as damage of cortical vs. subcortical cerebral structures. Indeed, clinical trials in acute ischemic stroke tend to prefer mRS 2 or better at 3 months as a cut-off for good recovery whereas acute hemorrhagic stroke trials have used various measurements of mRS at 3^25^ and 6 months to estimate recovery.^26,27^ When using mRS for assessing stroke recovery that has occurred between the acute phase and later on, it is impossible or difficult to estimate the mRS in the acute phase because during this phase the patient is not in her/his normal environment, making it problematic for the patient to perform the more global assessment items included in mRS (Figure 3). It is therefore common to adjust the observed outcome mRS at later time points by including acute phase NIHSS as a measure of initial stroke severity and compensate for this in the analyses. Even though many studies have evaluated outcome using a dichotomous cut-off e.g. mRS 0–2 vs. mRS 3–6, it has been suggested that instead analyzing the mRS as an ordinal scale variable may provide increased statistical value^28,29^ and that weighting between the individual mRS values may be useful.^30^ On the other hand, an ordinal scale result may be difficult to interpret from a clinical practical point of view,^30^ even though this concern may be less important in studies trying to find genetic signals indicating proof-of-concept rather than directly clinically important differences.

**Figure 4.**
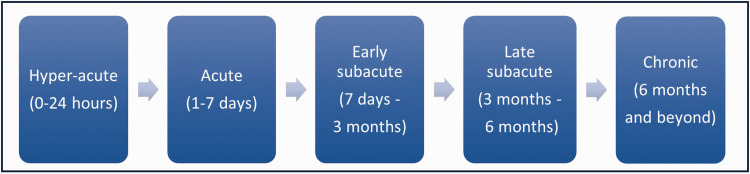
Time after stroke can be grouped into different phases. The Stroke Recovery and Rehabilitation Roundtable taskforce^15^ has suggested 5 different time phases after stroke. It should be noted that, in real life these phases may overlap, biological mechanisms are often present in more than one of the phases, and also differ between individual stroke patients.

An even more elementary group of parameters are biomarkers such as imaging methods and different omic analyses including proteomics and metabolomics in blood with a potential to demonstrate proof-of-concept of how genetic factors may influence stroke recovery (see below).

Several characteristics other than genetics influence recovery considerably after stroke. Initial stroke severity,^31^ sex,^31^ stroke mechanism,^31^ age,^31^ pre-stroke mRS,^31^ thrombolysis/thrombectomy treatment,^31^ diabetes,^32^ and good pre-treatment collaterals on angiography^33^ have been related to clinical outcome at 3–6 months. It can be mentioned that even though there is an inter-relation between age and sex because women as a group on average experience stroke later in life, age and sex may still remain as independent predictors for 3-month good functional outcome.^31^ In addition, other mechanisms post stroke may obscure the true recovery process. This includes recurrent stroke where genetic factors related to stroke risk rather than recovery may be important, and mortality occurring after the index stroke where concomitant diseases may be one driving factor.

### Outcome measures in the hyper-acute phase

Studies focusing on hyper-acute recovery occurring at 0–24 hours or slightly thereafter often use less complex assessments such as neurological body function and biomarkers. The NIHSS is often the basis for evaluation of neurological function and changes in this phase. The term ΔNIHSS_24h_ here denotes changes from baseline (within 6 h of stroke onset) to 24 h after acute ischemic stroke.^7^ A recent study identified eight genetic loci related to NIHSS variance at 24 hours explaining 1.8% of the ΔNIHSS_24h_ variability. Two of the loci indicated genes related to neuronal excitability, supporting that excitotoxicity may be related to brain injury in ischemic stroke in humans.^34^ By using the software tool genome-wide complex trait analysis,^35^ the authors also reported that 8.7% of the ΔNIHSS_24h_ variance was explained by common genetic variations, implying that additional, not yet identified genetic loci may be related to ΔNIHSS in acute ischemic stroke.^34^

Neuroimaging evaluation of cerebral edema and cerebrospinal fluid volume changes^36^ and hemorrhagic transformation^37^ can also be assessed as biomarkers of ischemic stroke outcome in the hyperacute phase.^36^ Other radiomic features may also be useful^32^ and machine learning can help performing imaging analyses in large cohorts.^38^

Analyses of blood biomarkers related to e.g. injury, repair, inflammation coagulation, endothelial, and blood-brain barrier function and how these are influenced by genetic factors can also serve as important intermediary outcome measures in the acute or later phases after stroke and provide important clues for identifying metabolic mechanisms involved post stroke. Recommendations for biological sample collection and storage has been published on behalf of the International Stroke Genetics Consortium.^39^ An updated version is being considered, and as mentioned above, harmonization of phenotype measures are also important to enable meta-analyses.^13^

## Genetic analyses in stroke recovery

There are several types of genetic analyses to consider for examining genetic influence on stroke recovery (Table 2). Most published studies have used candidate gene approaches, examining single nucleotide polymorphisms, a method that has been used since the beginning of the 21^st^ century for ischemic stroke^5^ and hemorrhagic stroke^6^ outcome studies. The candidate genes chosen for investigation have been selected from results from animal studies and theoretical hypothesis generation. However, there have been problems in independently confirming initially reported findings in candidate gene studies. A study of reported candidate gene findings for stroke risk showed that many of the published findings became non-significant when statistically adjusting for number of genes examined, were not significant in GWAS studies, and might suffer from publication bias.^40^ One candidate gene that has received substantial consideration regarding stroke recovery is the *BDNF* (brain-derived growth factor) gene. The rs6265 Val66Met variant in the *BDNF* gene has been examined in several studies.^7^ This variant has been investigated not only regarding general stroke recovery but also how it may influence the effect of different therapeutic rehabilitation measures. Studies of this variant from before 2016 have been addressed in a previous report.^4^ Studies since 2016 have continued to show both associations^12,41–49^ and no associations^12,50–52^ with stroke outcome or post-stroke treatment. Explanations for these discrepancies may be small number of subjects being investigated, varying case-mix regarding included patients in studies, and confounding factors not being accounted for. Different outcome measures and timing of evaluations may also influence the possible impact of the Val66Met variant. One observation that may be of special interest is that cognitive function after stroke may be related to the rs6265 *BDNF* variant,^49^ supported by a recent report that the rs6265 *BDNF* variant was associated with poorer cognition at 1 year after stroke onset.^12^ The number of subjects in different BDNF studies have varied considerably – as exemplified in Table 3. *ApoE* is another gene that has been repeatedly investigated both regarding ischemic^4,5,7,12,52^ and hemorrhagic stroke.^6^

**Table 2.** Different categories of genetic analyses that can be used for examining genetic influence on stroke recovery.

Type of genetic analysis	Example on outcome measure	Example reference
Candidate gene	mRS at 3 months or other time points	See reviews by Carnwath et al. (ischemic stroke)^5^ and Guo et al. (hemorrhagic stroke)^6^
GWAS	mRS at 3 months	Söderholm et al.^53^ and Mola-Caminal et al.^54^
Exome sequencing	Recurrent stroke	Xie et al.^55^
Whole genome sequencing	mRS at different time points	Cheng et al.^56^ and Chen et al.^76^
Genetic imbalance	mRS at 3 months	Pfeiffer et al.^60^
CNV	mRS at 3 months	Cole et al.^59^
Mendelian Randomization	mRS at 3 months	Gill et al.^77^

mRS indicates modified Rankin scale; GWAS: Genome Wide Association Study; CNV: Copy Number Variation.

**Table 3.** Examples of sample sizes in BDNF and APOE genetic studies on stroke outcome and recovery from 2016 and later.

Genetic Variation/Analysis	Study	n	Outcome	Comment
Val66Met (*BDNF*)	Cramer 2024^12^	489	Cognition at 1 year	
	Kim 2016^41^	42	Motor system function upper extremity	
	Kim 2016^42^	35	Motor function upper extremity	
	Helm 2016^43^	27	Adaption of walking pattern	
	Shiner 2016^44^	55	Motor function upper extremity	
	van der Vliet 2016^45^	80	Circuit tracing task	
	Fridricksson 2018^46^	67	Aphasia after tDCS treatment	
	Braun 2020^47^	829	mRS	
	Dresang 2022^48^	17	Aphasia	
	Han 2020^49^	86	Cognitive function	
	French 2018^50^	63	Functional mobility	No association
	Zhou 2019^51^	778	Acute functional outcome (mRS)	No association
	Cramer 2022^52^	216	Motor status	No association
*ApoE*	Cramer 2024^12^	515	Motor/functional outcomes	No association
	Cramer 2022^52^	216	Motor status	No association

*BDNF* indicates Brain-Derived Neurotrophic Factor; tDCS: transcranial Direct Current Stimulation; mRS: modified Rankin scale; *ApoE*: Apolipoprotein E.

Genome Wide Association Studies (GWAS) also focus on individual genetic single nucleotide polymorphisms (SNPs) but perform simultaneous agnostic analyses of a large number of selected SNPs throughout the genome. Because so many SNPs are analyzed at the same time, the statistical p value needs to be adjusted to multiple testing and the significance level is usually set to 5*10e-8. Two large studies have used this approach to detect SNPs related to stroke recovery. The Genetics of Ischaemic Stroke Functional Outcome (GISCOME) study (n = 6165) identified a SNP, rs1842681, in the intronic part of *LOC105372028* in chromosome 18 to be related to good vs. bad outcome defined as mRS 0–2 vs. mRS 3–6 at 3 months after ischemic stroke onset.^53^ It was suggested that the identified polymorphism could affect brain plasticity with implications for stroke recovery.^53^ The finding from the GISCOME study has recently been replicated in another study.^12^ The second large GWAS of stroke recovery, supported by the Genetic contribution to functional Outcome and Disability after Stroke (GODS) project also examined recovery according to mRS at 3 months (n = 1791 in a stringent meta-analysis). Only ischemic stroke patients with initial NIHSS severity >4 were included and patients with posterior vascular territory and lacunar strokes were excluded which contributed to obtaining a more homogenous sample. A SNP in the intronic region of the *PATJ* (Pals1-associated tight junction) gene in chromosome 1 was related to worse functional outcome. The protein encoded by the *PATJ* gene is found in epithelial cells and may be related to homeostasis and the blood brain barrier.^54^

Exome sequencing and whole genome sequencing carry promise to identify rare genetic variations related to stroke recovery and outcome. One study using whole-exome sequencing data from the UK Biobank detected no single rare variant related to stroke risk.^55^ However, a gene-based analysis accounting for loss-of function and deleterious missense variants with minor allele frequency below 0.01 that evaluated 16074 genes found that variations at *CYP2R1* in chromosome 11 were related to stroke risk.^55^ A subsequent validation analysis (n = 1706) showed that these variations at *CYPR2R1* were also related to stroke recurrence.^55^ Thus, the study demonstrated that analysis of rare variants by using exome sequencing data and gene-based analysis can identify rare genetic variants contributing to stroke outcome, in this case stroke recurrence. More studies using exome sequencing data for stroke recovery genetic research are likely to appear in the future. A whole genome sequencing study of over 10 000 Chinese ischemic stroke patients has been performed and outcome results are awaited.^56^

Structural genetic variants can be defined as DNA variants larger than 50 base pairs.^57^ Copy number variations (CNVs) constitute one type of structural DNA genetic variants^57^ where a segment of DNA can have different numbers of copies.^57,58^ Investigations on CNV and stroke recovery are ongoing.^59^ Genetic imbalance, defined as the number of protein-coding genes with copy number variations in a subject’s DNA has in a study of 3314 subjects been related to less favorable outcome after ischemic stroke.^60^

Another study also found results suggesting that genomic instability may be related to stroke outcome.^61^ The study examined a polygenic risk score for genetic variants (a score containing 127 variants from all somatic (non-sex) chromosomes except chromosome 21), predisposing to mosaic loss of Y (mLOY) (n = 6165) - which may gradually occur during aging - and stroke outcome at 3 months in the GISCOME cohort and found a significant association especially in women and also after adjustment for age. This suggests that genetic predisposition to mLOY in leukocytes may be a biomarker indicating a broader genomic instability also in other tissues in both sexes.^61^ A subsequent study examined 1323 men with ischemic stroke, not receiving acute recanalization therapy and defined mLOY to be present if this was detected in at least approximately 10%^62^ of genotyped cells. The study showed that mLOY in itself was associated with an increased risk for worse outcome, defined as mRS = 3 or more at 3 months after stroke onset.^63^ The authors suggested that mLOY could indicate genomic instability or influence the expression of genes involved in immunological functions in microglia and leukocytes in the brain, thereby affecting stroke recovery.^63^

Mitochondrial DNA copy number (mtDNA-CN) is the number of mitochondrial genomes per cell and can be seen as a biomarker of mitochondrial function. mtDNA-CN has been associated with increased rate of stroke recurrence, mortality after stroke and worse functional outcome after stroke in a study of 10241 stroke patients.^64^

Mendelian Randomization studies for stroke outcome have increased considerably in number of publications during the last few years. A PubMed search on Dec 8, 2024, with the term: *“stroke”[Title] AND (“outcome”[Title] OR “outcomes”[Title]) AND (“mendelian randomization”)* yielded 26 publications, the oldest from 2019 and no less than 16 publications from 2024. The increased number of published Mendelian Randomization studies in general has raised concerns regarding quality issues.^65^ Mendelian Randomization studies of good quality can deliver important insights into biological mechanisms but caution has been raised that it is imperative to avoid several possible pitfalls including e.g. horizontal pleiotropy and vertical pleiotropy.^65^

## Intervention trials in stroke recovery

Trials have examined if genetic variants may influence the effect of different therapeutic rehabilitation measures. The rs6265 Val66Met variant in the *BDNF* gene has been investigated in this context in stroke patients in several trials where the variant could be related to results from e.g. robot assisted arm motor therapy,^41^ split-belt treadmill walking,^43^ Wii based movement therapy or constraint-induced movement herapy,^44^ repeated motor skill learning with a circuit tracing task,^45^ transcranial direct current stimulation (tDCS) to the left hemisphere during behavioral aphasia treatment,^46^ and repetitive transcranial magnetic stimulation (rTMS) in patients with aphasia.^48^ The research field on genetic influence on rehabilitation measures after stroke is likely to expand in the future.

Genetic influence on pharmacological treatment after stroke is now becoming a reality for clinical practice. A review on pharmacogenomics in stroke was recently published and discussed genetic variants that influence the individual responses to aspirin, clopidogrel, warfarin, and statins.^66^ Treatment with clopidogrel is often used as secondary prevention after stroke and TIA. Clopidogrel is a prodrug which is metabolized to active drug by the CYP2C19 enzyme. Loss-of-function alleles in the *CYP2C19* gene can result in lower effect of clopidogrel medication. Loss-of-function alleles in *CYP2C19* are common and can occur in about 15%–20% in White and up to about 40%–60% in Asian inidviduals.^67,68^ A randomized double-blind placebo-controlled trial included 6412 patients with acute minor stroke or TIA who with rapid point-of care genotyping had been found to be carriers of *CYP2C19* loss-of-function alleles.^67^ The patients were randomized to treatment with either clopidogrel or ticagrelor and both groups in addition received aspirin for the first 21 days after their index event. After 90 days, there had been significantly more strokes in the clopidogrel group (7.6%) vs. the ticagrelor group (6.0%, p = 0.008). There was no significant difference for occurrence of severe or moderate bleeding between the groups.^67^ It should be pointed out that one limitation was that in the study the included patients were not compared to non-carriers of the CYP2C19 loss-of-function alleles.^67^ The National Institute for Health and Care Excellence (NICE) guidelines now suggests *CYP2C19* genotype testing to assess if clopidogrel is a suitable antiplatelet drug for people who have just had an ischemic stroke or a transient ischemic attack (TIA) (www.nice.org.uk/guidance/dg59). The American Heart Association has stated that *CYP2C19* genetic testing before prescription of clopidogrel or ticagrelor/prasugrel in patients with acute coronary syndrome or percutaneous coronary intervention can be beneficial.^68^ Other pharmacogenetic findings are under evaluation^66^ and new studies are underway including the ESTREL-PRECISION trial that aims at studying genetic profiles in relation to outcome and levodopa treatment.^69^

## Linking genomics to other omics

**Figure 5.**
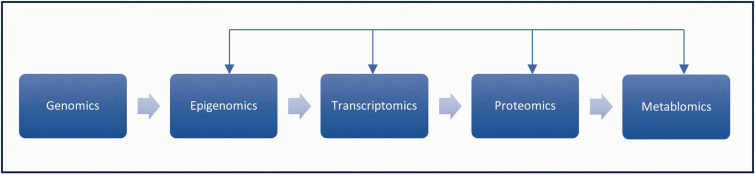
Genomics are related to other omics. The main pathway is from genomics onwards through the different omics to proteomics and metabolomics, but several other interactions and feed-back loops also occur. For more details, see other articles in this special issue.

The genome has influence on other omics (Figure 5).^70^ Not only the DNA content, but also how and when the DNA is translated for downstream effects is of fundamental importance. Epigenetic factors can influence gene expression and transcription through several mechanisms including DNA methylation, histone modifications, and micro-RNA regulation.^71^ An epigenome-wide association study analyzing blood samples obtained during the first 24 hours after ischemic stroke onset (n = 643 in the discovery, n = 62 in the replication cohort) found that the degree of DNA methylation of the CPG (“ 5'-C-phosphate-G-3') site cg00039070 in the body of the *EXOC4* gene on chromosome 7 was negatively correlated with the improvement according to NIHSS between baseline and hospital discharge.^72^ A similar study performed an epigenome-wide analysis of blood samples obtained during the first 24 hours after stroke onset (n = 316 in the discovery, n = 92 in the replication cohort) and detected one differentially methylated position and four differentially methylated regions that were related to stroke recovery, measured as mRS outcome at 3 months, indicating that DNA methylation analyses may provide insight into new stroke recovery mechanisms.^73^

## Future perspectives

New methods for genetic analyses are evolving at a fast pace and costs for analyses are declining. Interactions between different genes are often not accounted for, but polygenic scores may be one way to address this. Machine learning^74^ and Artificial Intelligence will provide means for studying such interactions and enable analyses of large data sets that have until now been very difficult or impossible to perform. However, a critical, clinical understanding including careful, adequate phenotyping is still needed as has e.g. been discussed regarding Mendelian Randomization.^65^ Genetic findings in human studies will often subsequently need to be examined more closely in experimental studies to provide increased insight into biological mechanisms influencing stroke recovery.^7^ Individualized treatment is already at hand regarding pharmacogenomics and is likely to evolve also for other personalized therapies in the future.

## Conclusions

Stroke recovery genomics encompasses many fields where timing, type of cerebral injury, and type of recovery vary considerably. Depending on what aspect on stroke recovery that is to be studied, the outline of a study is very different and should be carefully planned in advance before start-up. Different approaches should be designed depending on whether acute phase recovery or long phase recovery is the focus, but in all cases detailed phenotyping according to published recommendations^13^ is essential to enable harmonization of large cohorts for multi-center studies. Pre-stroke clinical information is needed to evaluate true recovery after an index stroke. To detect proof-of-principle signals, focus on specific, detailed outcome measures may be more rewarding than broader general outcome measures such as mRS which should nonetheless also be included to allow for comparisons with other studies. Adjustment for other non-genetic factors related to stroke recovery is necessary. New analytical methods including genome sequencing and new computer analytical method hold promise for providing new insights into the biology of stroke recovery and thereby identifying individual personalized treatments.

## Disclosures

AG Lindgren has served as national leader for Sweden and Denmark for the NAVIGATE trial and as national leader for Sweden for the PACIFIC-STROKE trial. He is national leader for Sweden for one ongoing stroke trial, and local PI for the StrokeCLOSE study. He is Co-chair for the Global Alliance for International Stroke Genetics Consortium Acute and Long-term Outcome studies. He is Co-PI for The Genetics of Ischaemic Stroke Functional Outcome (GISCOME) study. He reports personal fees from Arega, Bayer, Astra Zeneca, BMS Pfizer, and Novo Nordisk.
